# *Boesenbergia rotunda* and Its Pinostrobin for Atopic Dermatitis: Dual 5-Lipoxygenase and Cyclooxygenase-2 Inhibitor and Its Mechanistic Study through Steady-State Kinetics and Molecular Modeling

**DOI:** 10.3390/antiox13010074

**Published:** 2024-01-05

**Authors:** Desy Liana, Chatchakorn Eurtivong, Anuchit Phanumartwiwath

**Affiliations:** 1 College of Public Health Sciences, Chulalongkorn University, Bangkok 10330, Thailand; 6478303153@student.chula.ac.th; 2 Department of Pharmaceutical Chemistry, Faculty of Pharmacy, Mahidol University, Ratchathewi, Bangkok 10400, Thailand; chatchakorn.eur@mahidol.ac.th

**Keywords:** fingerroot, flavonoid, anti-inflammatory, antioxidant, antibacterial, GC–MS

## Abstract

Human 5-lipoxygenase (5-LOX) and cyclooxygenase-2 (COX-2) are potential targets for suppressing pruritic skin inflammation in atopic dermatitis (AD). In addition, *Staphylococcus aureus* colonization and oxidative stress worsen AD skin conditions. We aimed to investigate anti-inflammatory activity, using 5-LOX and COX-2 inhibitions, and the anti-staphylococcal, and antioxidant potentials of several medicinal plants bio-prospected from traditional medicine related to AD pathogenesis. Essential oils and hexane fractions were prepared and analyzed using gas chromatography–mass spectrometry. *Boesenbergia rotunda* hexane extract displayed anti-*Staphylococcus aureus* (MIC = 10 µg/mL) and antioxidant activities (IC_50_ = 557.97 and 2651.67 µg/mL against DPPH and NO radicals, respectively). A major flavonoid, pinostrobin, was further nonchromatographically isolated. Pinostrobin was shown to be a potent 5-LOX inhibitor (IC_50_ = 0.499 µM) compared to nordihydroguaiaretic acid (NDGA; IC_50_ = 5.020 µM) and betamethasone dipropionate (BD; IC_50_ = 2.077 µM) as the first-line of AD treatment. Additionally, pinostrobin inhibited COX-2 (IC_50_ = 285.67 µM), which was as effective as diclofenac sodium (IC_50_ = 290.35 µM) and BD (IC_50_ = 240.09 µM). This kinetic study and molecular modeling showed the mixed-type inhibition of NDGA and pinostrobin against 5-LOX. This study suggests that *B. rotunda* and its bioactive pinostrobin have promising properties for AD therapy.

## 1. Introduction

Atopic dermatitis (AD) is a chronic inflammatory skin disease with high prevalence worldwide. People with this skin disorder not only experience pathogenic symptoms such as inflammation, redness, itching, infection, and scaling but they may also suffer from a lower quality of life, mental burden, and depression [1]. AD is caused by defects of the epidermal skin barrier and dysregulation of the immune system. The pathophysiology of AD is known to be multifactorial, influenced by genetic and environmental factors [2]. The defect of the skin barrier is caused by several factors, including microbial infection, low temperature, humidity, and chemical exposure. Genetic factors such as the mutation of the filaggrin gene may also affect the defect of the skin barrier. This common skin disease is known to develop in 85% of patients before the age of 5 years and may persist into adulthood. A family history of this disease increases the risk of developing AD. Reduction in the skin microbiome (e.g., *Proteobacteria*, *Cyanobacteria*, *Actinobacteria*) in patients with AD and colonization of *Staphylococcus aureus* are known to affect the severity of the disease [3]. *S. aureus* colonization has been reported in more than 90% of patients [4]. Dominancy of *S. aureus* in the skin microbiome and several environmental stimuli may worsen symptoms of AD. Exposure to ultraviolet light leads to the generation of reactive oxygen species, resulting in oxidative stress, which is also involved in inflammation, itch, and skin barrier impairment [4].

Topical corticosteroids are used as first-line drugs for AD therapy due to their efficacy in controlling skin disorders as potent anti-inflammatory drugs [5]. Corticosteroids suppress inflammation by targeting phospholipase A_2_ in the arachidonic acid pathway [6]. However, the prolonged use of topical corticosteroids may cause undesirable side effects, including dermal atrophy, striae, hypopigmentation, rosacea, telangiectasia, purpura, stellate pseudoscars, hyperpigmentation, and local irritation [7]. Because of the serious side effects of topical corticosteroids, complementary and alternative treatments for AD therapy have been used in recent years [8]. Approximately 50% of patients with AD have reported using complementary medicines such as traditional Chinese medicine (TCM), traditional Malay medicine (e.g., cupping, massage), aromatherapy, vegetable oils (e.g., olive oil, virgin coconut oil, omega oil, blackseed oil), homeopathy, and dietary therapy (e.g., prebiotic, probiotic, vitamins) [7,9]. A low intake of antioxidant-related nutrients such as dietary vitamin E and beta-carotene was found to be associated with a higher risk of AD [10]. Accordingly, nutraceutical and functional foods derived from natural products show promise as dietary supplements [11]. Studies have shown that the dietary intake of fermented foods during pregnancy lowers the risk of infantile AD [12]. In addition, increased consumption of *Lactobacillus rhamnosus* probiotics, retinol, and minerals such as calcium and zinc was also shown to reduce the risk of AD in childhood [12].

Numerous medicinal plants are used in traditional herbal medicine to manage skin diseases, including *Artemisia vulgaris*, *Boesenbergia rotunda*, *Camellia sinensis*, *Cassia fistula*, *Dimocarpus longan*, *Mentha villosa*, and *Wedelia trilobata*. In TCM, *C. sinensis* is used as a bioactive constituent in skin care [13]. The powder of *D. longan* by-products such as peels and seeds has been used to treat eczema [14]. The rhizome of *B. rotunda* is used to treat dermatitis, wounds, and relieve skin itchiness [15,16]. In Ayuverdic medicine, *C. fistula* has been used to treat eczema [17]. *W. trilobata* has been used traditionally by some cultures to treat wounds [18]. Furthermore, Mentha species is commonly used directly on the skin as an analgesic and cooling agent [19]. In Western and Eastern cultures, in particular Morocco, the powder from the leaves of *M. villosa* is used to treat skin diseases [20]. On the other hand, *A. vulgaris* can be used to treat burns and ear pain [21]. This demonstrates that they may have pharmacological benefits in the treatment of inflammatory skin diseases such as AD.

**Figure 1:**
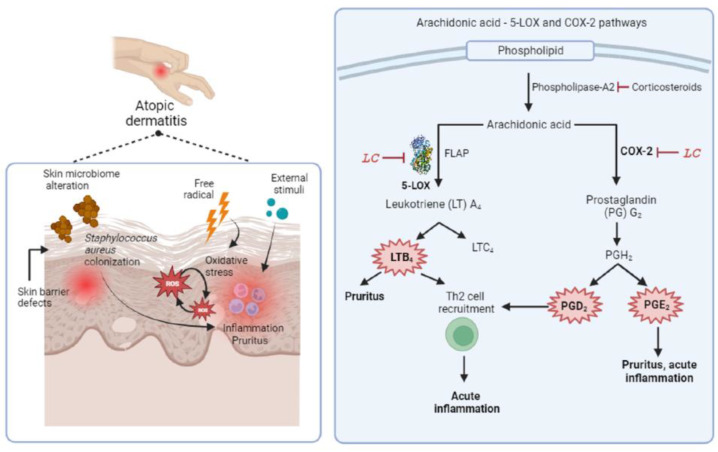
Atopic dermatitis is a multi-facet inflammatory skin condition characterized by skin microbiome alteration by colonization of *S. aureus*, oxidative stress, skin barrier defects, and chronic pruritic inflammation. Several proinflammatory mediators are found to be abundant in AD skin compared to normal skin, including LTB_4_, PGD_2_, and PGE_2_. The suppression of acute skin inflammation and pruritus can be done by targeting 5-LOX and COX-2 pathway; LC: lead compound, FLAP: 5-LOX activating protein.

AD is caused by various mechanisms, including inflammation, oxidative stress, infection, skin barrier disruption, pruritus, and lesion. Hence, the potential of phyto-active ingredients derived from plant extracts for improving AD skin conditions can be assessed through various approaches, including anti-inflammatory, antioxidant, antimicrobial, wound-healing, and analgesic properties [22]. The polar fractions of medicinal plants are widely known to possess greater antioxidant activity, whereas nonpolar fractions show greater antibacterial activity. Hence, in this study, we purposely selected nonpolar fractions, which may be able to penetrate easily into the cell membrane. Furthermore, evaluations of nonpolar antioxidative agents have been relatively limited to date. Compared to normal skin, leukotriene B_4_ (LTB_4_) has been found to be higher in AD skin, and it has been implicated in the recruitment of T helper 2 (Th2) cells and neutrophils, leading to acute inflammation. Accordingly, the suppression of LTB_4_ production via the inhibition of 5-lipoxygenase (5-LOX) in the arachidonic acid pathway is promising for AD treatment [23]. In addition, 5-LOX inhibitors were reported to reduce itch by suppressing LTB_4_ and substance P-induced itch and hence can be used as antipruritic agents in AD therapy [24,25]. A topical 5-LOX inhibitor, Q301 (zileuton), has been studied in a phase 2A clinical trial and found to improve AD skin lesions [26]. Moreover, pro-inflammatory prostanoids such as prostaglandin D_2_ (PGD_2_) and prostaglandin E_2_ (PGE_2_) were found to be abundant in AD skin compared to healthy skin. [27]. PGD_2_ is a Th2 cell recruitment mediator that is produced by allergen-stimulated mast cells [28]. As shown in Figure 1, the production of these prostanoids can be suppressed by inhibiting cyclooxygenase 2 (COX-2) as one of strategies for suppressing skin inflammation in AD [29,30].

Due to the serious adverse effects of topical corticosteroids, there is an urgent need for alternative therapies for AD. The promising multi-pharmacological action of herbal medicine prompted us to investigate the potential of nonpolar extracts derived from seven medicinal plants bio-prospected from traditional medicine used to treat skin inflammatory disorders for AD therapy. Bioactivities related to the pathogenesis of AD such as antibacterial against *S. aureus*, antioxidant potential, and anti-inflammatory properties by 5-LOX and COX-2 inhibitions were evaluated. Furthermore, we performed bioactive compounds profiling of the bioactive extracts using gas chromatography–mass spectrometry (GC–MS). Bioactive compound isolation and a more thorough assessment of the anti-inflammatory potency of the most promising extract were conducted. Eventually, mechanistic insight of the active compound through anti-inflammation was determined by enzyme inhibition kinetics and molecular modeling.

## 2. Materials and Methods

### 2.1. Plant Materials and Chemicals

The plant materials were collected from various sources in Thailand from November 2021 to January 2022. All plants species were identified and authenticated by a botanist. The voucher specimen numbers, including *A. vulgaris* L. [Asteraceae; 17531 (BCU)], *B. rotunda* (L.) Mansf. [Zingiberaceae; 17530 (BCU)], *C. fistula* L. [Fabaceae; 17528 (BCU)], *M. villosa* Huds. [Lamiaceae; 17532 (BCU)], and *W. trilobata* (L.) Hitchc. [Asteraceae; 17529 (BCU)], except for *C. sinensis* (L.) Kuntze [Theaceae; plant identification] and *D. longan* Lour. [Sapindaceae; plant identification], were deposited at the Department of Botany, Faculty of Science, Chulalongkorn University, in Bangkok, Thailand.

All commercially available chemicals and solvents were purchased from commercial companies and used without further purification. Quercetin hydrate (≥95% purity), gallic acid (≥97.5% purity), sodium nitroprusside (SNP) dihydrate (≥99% purity), sulfanilamide (>98% purity), *N*-(1-naphthyl) ethylenediamine dihydrochloride (>98% purity), and tetracycline hydrochloride (≥95% purity) were purchased from Sigma Aldrich, Darmstadt, Germany. Human recombinant COX-2 was purchased from Sigma Aldrich, Darmstadt, Germany. Human recombinant 5-LOX was purchased from Cayman Chemical, Ann Arbor, MI, the United States. In addition, arachidonic acid (>98% purity), *N*,*N*,*N*′,*N*′-tetramethyl-*p*-phenylenediamine (>98% purity), betamethasone dipropionate (>97% purity), nordihydroguaiaretic acid (>97% purity), and diclofenac sodium (>98% purity) were purchased from Tokyo Chemical Industry, Tokyo, Japan. Hexane as an analytical reagent (AR)-grade solvent, and methanol as a high-performance liquid chromatography-grade solvent (>99% purity), were purchased from RCI Labscan, Bangkok, Thailand.

### 2.2. Extraction of Essential Oils by Hydro Distillation

Each fresh and clean plant material (100 g) was hydrodistilled using a Clevenger apparatus (Betchai Bangkok Equipment and Chemical Co., Ltd., Nakhon Pathom, Thailand) and further extracted for 5 h according to the methods of the British Pharmacopoeia (BP, 1970) [31]. The ratio of plant material to distilled water was determined as 1:6. The extraction was performed repeatedly to obtain an adequate amount of essential oils (EOs). A tiny amount of EOs produced by certain plant species (e.g., *C. sinensis* and *D. longan* peels) was obtained by hydro distillation, followed by the extraction of hydrosol using ethyl acetate and subsequent evaporation. The obtained EOs were dried using anhydrous sodium sulfate. Each EOs was kept in a dark vial and stored at −20 °C until further analysis. The percentage yield of each EOs was calculated by Equation (1).

(1) $$\%\;\mathrm{yield}=\frac{\mathrm{weight}\;\mathrm{of}\;\mathrm{extract}\;\left(g\right)}{\mathrm{weight}\;\mathrm{of}\;\mathrm{fresh}\;\mathrm{plant}\;\mathrm{material}\;\mathrm{used}\;\left(g\right)}\times 100$$

### 2.3. Ultrasound-Assisted Extraction (UAE)

Each fine and dried powder of the plant material (50 g) was soaked in n-hexane (250 mL) and then subjected to ultrasound by placing the extract into a sonication bath at a frequency of 35 kHz, power of 100 W, and temperature of 25 °C. Ultrasound was performed for 30 min. The mixture was subsequently further macerated at room temperature for 24 h, and sonication was reapplied by renewing the solvent until the successive extraction was obtained. The percentage yield of each extract was calculated by Equation (2).

(2) $$\%\;\mathrm{yield}=\frac{\mathrm{weight}\;\mathrm{of}\;\mathrm{extract}\;\left(g\right)}{\mathrm{weight}\;\mathrm{of}\;\mathrm{dried}\;\mathrm{plant}\;\mathrm{material}\;\mathrm{used}\;\left(g\right)}\times 100$$

### 2.4. Isolation and Structure Elucidation of Pinostrobin

Pinostrobin, as a flavonoid, was non-chromatographically isolated by a precipitation method using a co-solvent mixture of n-hexane and ethyl acetate, obtaining off-white fine crystals of pinostrobin. Firstly, a crude *B. rotunda* hexane extract obtained from the UAE technique was suspended in the co-solvent mixture of n-hexane/ethyl acetate (3.0:0.2 *v*/*v*), resulting in the direct precipitation of off-white crystals. The off-white crystals were purified by several cycles of rinsing with tiny amounts of n-hexane. All purified crystals were collected and air-dried overnight before spectroscopic analysis. The percentage yield of the isolated compound was calculated from the dried rhizome weight, and the compound purity was determined using thin-layer chromatography. Furthermore, ultraviolet–visible (UV–Vis) spectrophotometry, GC–MS, and proton nuclear magnetic resonance (^1^H-NMR) techniques were used to identify and elucidate the structure of pinostrobin.

(3) $$\%\;\mathrm{yield}=\frac{\mathrm{weight}\;\mathrm{of}\;\mathrm{crystal}\;\left(g\right)}{\mathrm{weight}\;\mathrm{of}\;\mathrm{dried}\;\mathrm{plant}\;\mathrm{material}\;\mathrm{used}\;\left(g\right)}\times 100$$

### 2.5. Gas Chromatography–Mass Spectrometry Analysis

Volatile components of EOs and hexane extracts were analyzed using Finnigan Trace GC Ultra (Thermo Fisher Scientific, Waltham, MA, USA) equipped with a Phenomenex Zebron ZC-5 MS (Phenomenex, Torrance, CA, USA) capillary column (5% phenyl, 95% methyl poly siloxane, 30 m × 0.25 mm × 0.25 µm film thickness) and a DSQ quadrupole mass detector (Thermo Fisher Scientific, Waltham, MA, USA). The volatile components were further detected using MS with an electron impact ionization positive mode at 70 eV. The temperature of the electron ionization ion source and injection port was maintained at 230 and 240 °C, respectively, whereas the column’s initial temperature was kept at 60 °C for 3 min and then further increased 3 °C/min until it reached 240 °C. Helium (>99% purity) was used as a carrier gas with a flow velocity of 1 mL/s. A sample dissolved in methanol (1 μL, concentration = 1 mg/mL) was injected with a Finnigan Autoinjector AI3000 (Thermo Fisher Scientific, Waltham, MA, USA) with split ratio of 1:10. The detection of the components was performed by comparing the MS spectra sample with the NIST05 and Adams EO library. The percentage abundance of each identified component was determined based on the percentage area normalization method.

### 2.6. Antibacterial Activity

The antibacterial activity of all the extracts was initially screened by an agar disk diffusion assay. The European Committee for Antimicrobial Susceptibility Testing of the European Society of Clinical Microbiology Infectious Diseases was used as the standard guideline [32]. An exponential phase with optical density at 600 nm (OD_600_) values of approximately 0.5) of a methicillin-sensitive strain of *S. aureus* was obtained by culturing in a culture medium (20 mL) containing Luria agar (LA) Bactoagar and Lysogeny broth (LB) (1:1). Furthermore, the bacterial cell suspension (50 µL) was spread on LA Bactoagar and further incubated at 37 °C for 24 h. The zone of inhibition of both plant EOs and extracts was measured, and a minimum inhibitory concentration (MIC) value was further determined for only promising EOs/extract.

The MIC values were determined using a broth dilution assay. Tetracycline used as an antibiotic was dissolved in 50% ethanol while EOs, extracts, and pinostrobin dissolved in DMSO (0.2% *v*/*v*) were suspended in an LB medium to obtain the final concentrations of EOs/extract, pinostrobin, and tetracycline in the range of 0.1562–200 µg/mL. Then, 20 µL of the bacterial culture with an OD_600_ value of ~0.5 was taken into each tube, which was continuously inoculated. Afterward, the treated culture was incubated at 37 °C for 24 h in a shaking condition (180 rpm). The absorbance was further measured at 600 nm. An LB medium and 0.2% DMSO were used as positive controls, while the cell culture without treatment in an LB medium was used as a negative control. The experiments were performed in triplicate.

### 2.7. Determination of Antioxidant Activities

#### 2.7.1. 2,2-Diphenyl-1-Picrylhydrazyl Radical (DPPH) Scavenging Activity

Each extract/EOs sample (20 µL) at various concentrations (6.25–5 mg/mL) was placed to a 96-well plate, followed by the addition of 120 µM DPPH solution in methanol (180 µL). The plate was subsequently incubated for 30 min in the dark at 37 °C. After that, the absorbance was measured by a microplate reader at 517 nm. Quercetin and solvent were used as a positive control and a control, respectively. The radical scavenging percentage inhibition against DPPH was calculated using Equation (4).

(4) $$\%\;\mathrm{Inhibition}=\frac{\mathrm{Absorbance}\;\mathrm{control}-\mathrm{Absorbance}\;\mathrm{sample}}{\mathrm{Absorbance}\;\mathrm{control}}\times 100$$

#### 2.7.2. Nitric Oxide (NO) Radical Scavenging Activity

The technique was adapted and modified from the study of Thadhani et al. [33]. The Griess reaction was used to determine the NO radical scavenging activity. The NO radicals were generated from 10 mM sodium nitroprusside (SNP) in phosphate-buffered saline (PBS), pH 7.3. A range of sample concentrations (10 µL, 0.625–5 mg/mL) were reacted with 10 mM SNP in PBS at pH 7.3 (90 µL) at room temperature for 90 min in a 96-well plate under visible polychromatic light. Afterward, 1% sulfanilamide in 5% H_3_PO_4_ (50 µL) was added to the preincubated extract, followed by subsequent incubation in a dark condition with ambient temperature for 5 min. Following that, 50 µL of 0.1% *w*/*v N*-(1-naphtyl)-ethylenediamine (NED) in distilled water was added to each well before incubating at room temperature for 30 min in a dark condition. The diazotization of sulfanilamide with a nitrite ion occurred, followed by a coupling reaction with NED solution to generate a magenta-colored chromophore. The absorbance was measured using a microplate reader at 540 nm. Quercetin was used as a positive control. The nitrite level of the SNP-treated group was used as a control group to calculate the percentage of NO radical inhibition. A solvent was used as a control. The percentage inhibition was calculated using Equation (5).

(5) $$\%\;\mathrm{Inhibition}=\frac{\mathrm{Absorbance}\;\mathrm{control}-\mathrm{Absorbance}\;\mathrm{sample}}{\mathrm{Absorbance}\;\mathrm{control}}\times 100$$

#### 2.7.3. Ferric Reducing Antioxidant Power (FRAP)

Each extract/EOs sample (10 µL) was added to each well of a 96-well plate at various concentrations (1–5 mg/mL), followed by a freshly prepared FRAP reagent (190 µL). The mixture was then incubated at room temperature for 4 min. Vivid blue resulted from the reduction in ferrous ions into ferric ions. The absorbance was further measured at 595 nm. The FRAP value (Equation (6)) was determined by calculating the concentration of ferric ions in the samples relative to the calibration curve of FeSO_4_ (FRAP value in µM FeSO_4_/mg dry weight of extract).

(6) $$\mathrm{FRAP}\;\mathrm{value}=C\;\times \frac{v\;\left(\mathrm{mL}\right)}{d\;\times \;c\;\left(\frac{\mathrm{mg}}{\mathrm{mL}}\right)}$$

where C is the calibrated sample concentration, v is the volume sample, d is the dilution factor, and c is the concentration of the tested sample.

#### 2.7.4. Total Phenolic Content (TPC) Determination

Each extract/EOs sample (50 µL) was added to each well of a 96-well plate at various concentrations (1–2 mg/mL), followed by the addition of 10% Folin–Ciocâlteu reagent (50 µL). A 20 min incubation in the dark at room temperature was then performed, followed by the addition of a solution of 7.5% sodium carbonate (50 µL) and another 20 min incubation. The absorbance was measured at 756 nm using a microplate reader. TPC values were determined in accordance with a standard curve of gallic acid (mg gallic acid/g dry weight of extract).

#### 2.7.5. Total Flavonoid Content (TFC) Determination

Each extract/EOs sample (50 µL) at various concentrations (1–5 mg/mL) was added into every well of a 96-well plate, followed by the addition of 10% aluminum chloride solution (10 µL). After that, 150 µL of ethanol and 10 µL of 1 M sodium acetate solution were added, and the mixture was incubated for 40 min in the dark at room temperature. The absorbance was measured using a microplate reader at 415 nm, and the results of the TFC values were then interpreted as mg quercetin/g dry weight of the extract.

### 2.8. In Vitro Anti-Inflammatory Activities

Anti-inflammatory activities of selected bioactive plant extracts/EOs based on anti-staphylococcal and antioxidant activities were performed using COX-2 and 5-LOX inhibition assays.

#### 2.8.1. Cyclooxygenase-2 Inhibitory Assay

This method was adapted and modified from Chew et al. (2018) [34]. Human recombinant COX-2 expressed in insect cells (Sigma Aldrich, C0858) was used in this experiment. Initially, a solution of 100 mM Tris HCl buffer, pH 8, was plated out in each well of a 96-well plate. Various concentrations of 40 µL samples (dissolved in polysorbate-20 and DMSO) were further added to the plate, followed by the addition of 40 µL of 40 U COX-2 enzyme (in ice-cool 100 mM Tris HCl buffer, pH 8). The mixture was incubated at 25 °C for 15 min under dark condition. Following the incubation, 10 µL of 20 mM *N*,*N*,*N′*,*N′*-tetramethyl-*p*-phenylenediamine (TMPD) and 4 µL of 10 µM of arachidonic acid were added to the mixture. Incubation of the mixture was further performed at a subdued light condition for 15 min. Prostaglandin G_2_ (PGG_2_) as an intermediate peroxide product was formed by the reaction and further oxidized the TMPD to a purple-colored TMPD complex. The absorbance was measured at 611 nm. The experiments were performed in duplicate wells. Diclofenac sodium was used as a positive control. The percentage of inhibition was calculated by Equation (7), and the IC_50_ values were determined.

(7) $$\%\;\mathrm{Inhibition}=\frac{\mathrm{Absorbance}\;\mathrm{control}-\mathrm{Absorbance}\;\mathrm{sample}}{\mathrm{Absorbance}\;\mathrm{control}}\times 100$$

#### 2.8.2. 5-Lipoxygenase Inhibitory Assay

We performed this method according to a previous study [35] with some modifications. Human recombinant 5-LOX (Cayman Chemical, E.C. 1.13.11.34, Ann Arbor, MI, USA) was used in this study. Various concentrations of 20 µL of the tested samples (dissolved in polysorbate-20 and DMSO) were plated in a 96-well UV transparent plate followed by the addition of 100 µL of 0.1 M potassium phosphate buffer, pH 6.3. The mixture was maintained at 25 °C before the addition of enzyme. We added 20 µL of 100 U 5-LOX enzyme (in 0.1 M potassium phosphate buffer, pH 6.3) to the sample solution followed by a 4 min incubation at 25 °C. Afterward, the reaction was initiated by adding 10 µL of 1 mM arachidonic acid as a substrate. The incubation was performed at 25 °C for 30 min, and the absorbance was measured at 234 nm using a microplate reader. The nonenzymatic control was used as a background. Nordihydroguaiaretic acid (NDGA) and betamethasone dipropionate (BD) were used as positive controls. The experiments were performed in two rounds to generate five replicate wells in total. The percentage of inhibition was calculated by Equation (8), and the IC_50_ values were determined.

(8) $$\%\;\mathrm{Inhibition}=\frac{\mathrm{Absorbance}\;\mathrm{control}-\mathrm{Absorbance}\;\mathrm{sample}}{\mathrm{Absorbance}\;\mathrm{control}}\times 100$$

#### 2.8.3. Steady-State Inhibition Kinetic Study of Pinostrobin to 5-Lipoxygenase

The steady-state kinetic study was performed to determine the mode of inhibition of the active compound to 5-LOX. The enzyme kinetic assay was performed using different concentrations of NDGA (0, 1.56, 3.125, and 6.25 µg/mL) and different concentrations of arachidonic acid (0, 8.33, 16.67, 33.33, and 66.67 µM). The same experimental conditions as described previously for determining enzyme inhibition were applied. The reaction was performed at 25 °C and measured kinetically for 30 min (BioTek Synergy H1 microplate reader, Winooski, VT, USA). A coefficient extinction of 23,000 M^−1^ cm^−1^ was used to determine the kinetics of the 5-LOX product, 5-hyroperoxyeicosatetraenoic acid (5-HETE) [36]. Furthermore, the enzymes’ velocity was plotted versus the substrate concentration, which was fitted to the Henri–Michaelis–Menten equation. In addition, the data were transformed to fit a double reciprocal plot using the Lineweaver–Burk method. The determination of the maximum velocity (V_max_, M/s), Michael constant (K_m,_ µM), and inhibitor constant (K_i_, µM) was carried out using GraphPad Prism v.9. All measurements were performed in two rounds of triplicate wells.

#### 2.8.4. Molecular Modeling of Pinostrobin to 5-Lipoxygenase

The ligands were constructed using Chem3D Pro 12.0; the chemical structures were drawn and energy-minimized using the MM2 [37] force field and saved in 3D format. The crystal structure of 5-LOX was obtained from the Protein Data Bank (PDB) [38] ID:6N2W [39] with resolution 2.71 Å. Discovery studio version 4.5 was used to prepare the crystal structure for docking, i.e., hydrogen atoms were added, and the crystallographic solvents and co-crystallized ligand were removed. Additionally, the iron cofactor was removed as there was no formation of chemical interaction with the NDGA co-crystallized ligand. The center of the 5-LOX binding site was defined at coordinates (x = 36.477, y = 65.271, z = 36.636) with a 10 Å radius. The basic amino acids lysine and arginine were defined as protonated. Furthermore, aspartic and glutamic acids were assumed to be deprotonated. The CS [40] scoring function was implemented for docking the ligands using the Genetic Optimization for Ligand Docking software package (GOLD) version 2022.3.0. Molecular docking was conducted at 100% efficiency at 50 docking runs per ligand.

### 2.9. Statistical Analysis

We determined whether there was a significant difference in the mean between groups via a one-way analysis of variance (ANOVA) with Duncan’s post hoc tests using IBM SPSS Statistics version 22.

## 3. Results

### 3.1. Preparation of Plant Extracts and Essential Oils

To prepare the essential oils (EOs) and hexane extracts, we performed hydrodistillation and ultrasound-assisted extraction of all seven medicinal plants, including *A. vulgaris*, *B. rotunda*, *C. sinensis*, *C. fistula*, *D. longan*, *M. villosa*, and *W. trilobata*, and all the percentage yields of EOs and extracts were determined (Supplementary Table S1). Among these medicinal plants, the rhizome of *B. rotunda* produced the highest yield of EOs. Consequently, the hexane extract derived from *B. rotunda* rhizome was oily, indicating the presence of EO components. On the other hand, *C. sinensis* leaves produced the highest yield of hexane extract.

### 3.2. Antibacterial Activity of Plant Extracts and Essential Oils

We performed the initial screening of antibacterial activity against *S. aureus* using an agar disk diffusion assay; among all the tested extracts, only *B. rotunda* extract exhibited the inhibition of *S. aureus* growth (9 mm), whereas the EOs derived from *B. rotunda*, *C. sinensis*, and *W. trilobata* exhibited antibacterial activity against *S. aureus* (9–13 mm) among the six plant EOs (Table 1). Based on our initial screening using the agar disk diffusion method, we continuously explored the determination of the MIC values of the *B. rotunda* extract and EOs. The results showed that the *B. rotunda* hexane extract (MIC = 10 µg/mL) was more active against *S. aureus* than its EOs (MIC = 100 µg/mL).

### 3.3. Antioxidant Potentials and Determination of Total Phenolic Content and Total Flavonoid Content

Among all the tested samples, both hexane extracts of *C. sinensis* and *B. rotunda* had the highest antioxidant potentials against DPPH radicals, while *D. longan* hexane extract exhibited the highest NO radical scavenging activity (Table 2). The *C. sinensis* and *B. rotunda* hexane extracts showed the highest DPPH radical scavenging activity, with IC_50_ values of 208.90 ± 4.81 and 557.97 ± 27.86 µg/mL, respectively, followed by *D. longan* peel hexane extract (IC_50_ = 1123.00 ± 115.21 µg/mL). On the other hand, the hexane extract of *D. longan* exhibited the highest NO radical scavenging activity (IC_50_ = 675.70 ± 54.37 µg/mL) compared with all the extracts and EOs tested. The EOs derived from *B. rotunda* were more active against NO radicals than *W. trilobata* EOs, with IC_50_ values of 1695.00 ± 128.18 and 2849.67 ± 82.80 µg/mL, respectively. According to the FRAP, we found that both *D. longan* peel EOs and *C. sinensis* hexane extract had relatively high reducing potentials, with values of 1.09 ± 0.11 and 3.87 ± 0.16 mM FeSO_4_/g extract, respectively, among all the tested samples.

**Figure 2:**
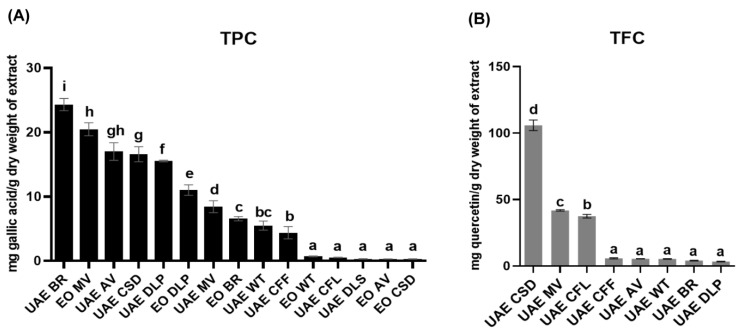
Determination of (**A**) total phenolic content (TPC) and (**B**) total flavonoid content (TFC) values of all tested samples. Hexane extract: ultrasound-assisted extraction (UAE); essential oils (EOs); *B. rotunda* (BR); *C. sinensis* (CSD); *C. fistula* leaves (CFL); *C. fistula* pods (CFF); *M. villosa* leaves (MV); *D. longan* peels (DLP); *D. longan* seeds (DLS); *W. trilobata* leaves (WT); and *A. vulgaris* (AV). Note: The TFC values of UAE DLS and all EOs were not determined because they were inactive at the highest tested concentration (5 mg/mL). One-way ANOVA was performed to compare the mean of each sample in each assay, and different letters indicate the significant difference of the mean.

According to the total phenolic content measurement, the *B. rotunda* hexane extract showed the highest content of phenolic compounds (24.335 ± 0.960 mg gallic acid/g dry weight of extract) among all the samples (Figure 2A and Supplementary Table S2). On the other hand, *C. sinensis* hexane extract had the highest total flavonoid content (105.904 ± 4.027 mg quercetin/g dry weight of extract) (Figure 2B and Supplementary Table S3).

### 3.4. Gas Chromatography–Mass Spectrometry Analysis of Phytochemicals

GC–MS analysis was performed to identify the volatile components present in bioactive extracts including *B. rotunda* hexane extract and EOs, *W. trilobata* EOs, and *C. sinensis* EOs. The major volatile components detected in the hexane extract derived from *B. rotunda* were composed of nerol (32.76%), pinostrobin (21.21%), β-cis-ocimene (8.76%), methyl cinnamate (5.46%), and 1.8-cineole (4.80%). On the other hand, camphor (35.25%) was mainly presented in *B. rotunda* EOs, followed by 1.8-cineole (20.47%), nerol (13.86%), camphene (4.97%), and methyl cinnamate (11.7%) (Supplementary Figure S1 and Table S4).

According to our results, *C. sinensis* EOs were mainly composed of n-hexadecanoic acid (52.54%) and linolenic acid methyl ester (21.98%), followed by phytol (11.28%) and linalool (2.72%) (Supplementary Figure S2 and Table S5). This is supported by the waxy characteristic appearance of distillable EOs. On the other hand, we found that spathulenol (16.97%) was the main constituent of the *W. trilobata* EOs, followed by germacrene D (12.5%), β-caryophyllene (9.88%), and junenol (7.89%) (Supplementary Figure S3 and Table S6).

### 3.5. Human Recombinant Cyclooxygenase-2 Inhibitory Activity of B. rotunda Essential Oils, Hexane Extract, and Pinostrobin

**Figure 3:**
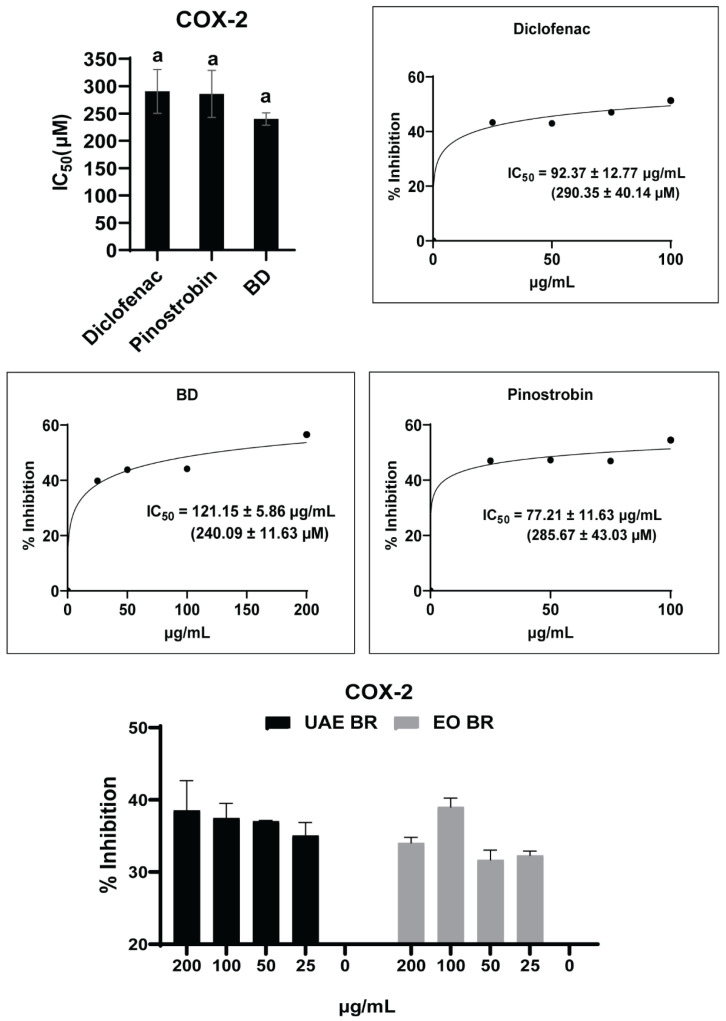
COX-2 inhibitory activity of *B. rotunda* hexane extract (UAE BR), essential oils (EOs BR), and pinostrobin compared to two standard drugs; NSAIDs diclofenac sodium and corticosteroid betamethasone dipropionate (BD). One-way ANOVA was performed to compare the mean of each sample in each assay. The same letter (a) indicated that the comparison between groups was not significant.

A previous report showed that racemic pinostrobin was more selective for COX-2 than COX-1. The racemic pinostrobin was comparable to etodolac as a positive control [41]. Accordingly, we evaluated the potency of *B. rotunda* EOs, hexane extract, and its isolated pinostrobin against COX-2 in this study. As shown in Figure 3, *B. rotunda* hexane extract and EOs exhibited relatively modest inhibitory activity towards COX-2 with IC_50_ values which were greater than 200 µg/mL. Furthermore, the isolated pinostrobin (IC_50_ = 285.67 ± 43.03 µM) demonstrated COX-2 inhibitory activity which was similar to two standard drugs; non-steroidal anti-inflammatory drugs (NSAIDs), diclofenac sodium (IC_50_ = 290.35 ± 40.14 µM), and corticosteroid betamethasone dipropionate (BD; IC_50_ = 240.09 ± 11.63 µM) (Supplementary Table S7). There is no significant difference in those IC_50_ values against COX-2.

### 3.6. Human Recombinant 5-Lipoxygenase Inhibitory Activity of B. rotunda Essential Oils, Hexane Extract, and Pinostrobin, and Steady-State Inhibition Kinetic Study

**Figure 4:**
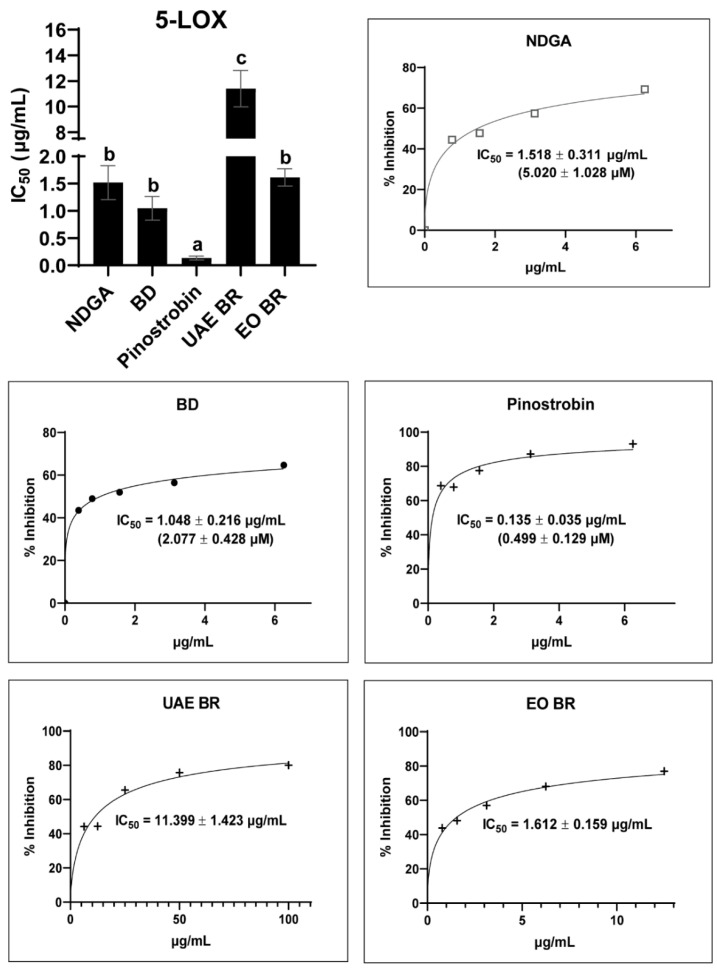
5-LOX inhibitory activity of *B. rotunda* hexane extract (UAE BR), essential oils (EOs BR), and pinostrobin compared to standard nordihydroguaiaretic acid (NDGA) and corticosteroid betamethasone dipropionate (BD). One-way ANOVA was performed to compare the mean of each sample in each assay, and different letters indicate the significant difference in the mean.

The anti-inflammatory potency induced by the 5-LOX inhibitory activity of *B. rotunda* EOs, hexane extract, and pinostrobin was also evaluated. The results showed that the *B. rotunda* hexane extract exhibited promising inhibitory activity toward 5-LOX while being 10-fold less potent than *B. rotunda* EOs. Interestingly, *B. rotunda* EOs had similar effectiveness with NDGA and BD. On the other hand, pinostrobin, a major bioactive compound found in the *B. rotunda* hexane extract, possessed 10-fold superior activity to the NDGA inhibitor and was 4-fold superior than BD as a standard AD drug (Figure 4 and Supplementary Table S8). Our finding showed that pinostrobin exhibited its potency as a dual 5-LOX and COX-2 inhibitor by in vitro assays.

**Figure 5:**
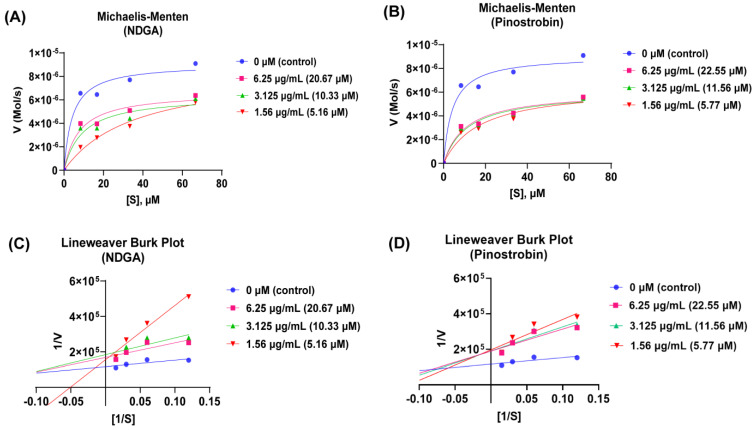
In vitro steady state kinetics of NDGA and pinostrobin using Michaelis–Menten and Lineweaver–Burk plots; (**A**) Michaelis–Menten plot of NDGA, (**B**) Michaelis–Menten plot of pinostrobin, (**C**) Lineweaver–Burk plot of NDGA, and (**D**) Lineweaver–Burk plot of pinostrobin.

Based on the Michaelis–Menten and Lineweaver–Burk methods, pinostrobin exhibited higher 5-LOX inhibition activity than NDGA (K_i_ pinostrobin < K_i_ NDGA), as shown in Supplementary Table S9. Based on both plots (Figure 5 and Supplementary Table S10), the type of inhibition for both NDGA and pinostrobin was shown to be the mixed-type inhibition (K_m_ increased, V_max_ decreased). NDGA is widely known as a redox-active inhibitor that is bound in the active site of 5-LOX, resulting in capping helix alteration [42]. However, limited information is available about its probability to bind to the allosteric site of 5-LOX. Furthermore, there is no report about the pinostrobin inhibitory action to 5-LOX. Molecular modeling was further carried out to support these findings.

### 3.7. Molecular Modeling of Pinostrobin and Nordihydroguaiaretic Acid to 5-Lipoxygenase

The co-crystallized ligand, NDGA, was removed from the 5-LOX protein crystal structure, energy-minimized, and docked back into the 5-LOX binding site. The lowest energy conformer was superimposed onto the co-crystallized NDGA, and the root-mean-square deviation (RMSD) was calculated for the heavy atoms using DockRMSD [43] (Supplementary Figure S8). The calculated RMSD value is relatively acceptable (RMSD = 2.424 Å); RMSD values of 2–3 Å are broadly acceptable due to conserved molecular orientations, and this is indicative that the docking protocol is relatively reliable [44].

**Figure 6:**
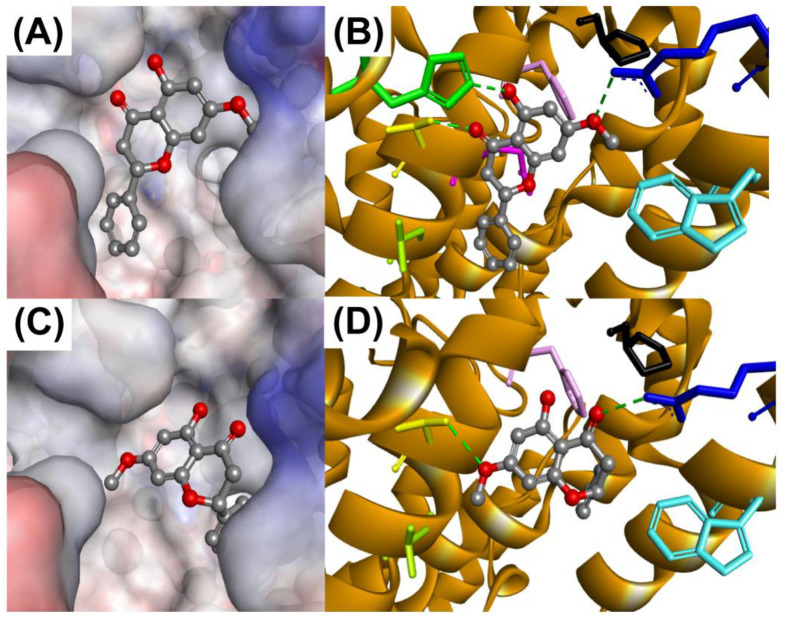
The predicted binding modes and interactions of (R)- (**A**,**B**) and (S)-pinostrobin (**C**,**D**) enantiomers with the 5-lipoxygenase binding site. The protein surfaces are rendered with blue and red depicting partially positive and negative charges, and neutral charge for grey areas (**A**,**C**). Amino acids predicted to form hydrogen bonding are depicted: His432 (green), Thr364 (yellow), Arg596 (blue), and Gln363 (violet). Residues predicted to form hydrophobic interactions: Phe359 (pink), Pro569 (black), Trp599 (turquoise), and Leu368 (lime green) (**B**,**D**).

Pinostrobin is a chiral compound and exists as two different enantiomers: (R)- and (S)-pinostrobin. Based on a previous study, pinostrobin isolated from *B. rotunda* was found to be racemic [45]. Thus, both enantiomers were docked into the 5-LOX binding site. The CS scores are relatively similar for both enantiomers, 25.27 and 25.62, for (R)- and (S)-pinostrobin, respectively, and were slightly higher than NGDA which scored 24.75, which may indicate stronger 5-LOX inhibition by pinostrobin. Based on the docking scores, it is predicted that both enantiomers are almost equally capable of inhibiting 5-LOX. The binding site is mainly hydrophobic and can form hydrophobic interactions with the inhibitors. When visualizing the binding modes of the highest scoring conformers, it was seen that they displayed different binding orientations, albeit similar interactions at the binding site (Figure 6).

## 4. Discussion

Based on the antibacterial activity, *B. rotunda* was the best bioactive medicinal plant against *S. aureus* among all the tested plants. The hexane extract of *B. rotunda* rhizome had an MIC value of 10 µg/mL, whereas its EOs had an MIC value against *S. aureus* of 100 µg/mL (Table 1). The *B. rotunda* hexane extract exhibited anti-staphylococcal activity, being 10-fold more active than its EOs. In addition, the previous report showed that the ethanolic extract derived from *B. rotunda* rhizome exhibited inhibition toward *S. aureus* with an MIC value of 310 µg/mL [46]. Similarly, the study reported by Teethaisong et al. [47] showed that *B. rotunda* rhizome ethanolic extract was active against β-lactam–resistant *S. aureus* with an MIC value of 16 µg/mL. As compared with these previous studies, our *B. rotunda* rhizome hexane extract possessing an MIC value of 10 µg/mL could be a promising dietary ingredient against *S. aureus*. The composition of volatile components presented (e.g., functional group, stereochemistry, and structural configuration) may affect the antibacterial activity of plant extracts/EOs [48]. The higher antibacterial activity of both *B. rotunda* rhizome EOs and hexane extract compared with other tested plants was possibly due to the presence of more bioactive components against the bacteria. The major component, nerol, and other components, including unsaturated cyclohexane such as terpineol (Supplementary Figure S1 and Table S4), are recognized as potent antibacterial agents [48].

On the other hand, the moderate antibacterial activity of the *C. sinensis* EOs may be caused by the integration of fatty acids as the major compounds found in *C. sinensis*. Fatty acids may penetrate into the bacterial cell membrane, leading to the rupture of bacterial cells. Fatty acids possess antibacterial activity due to their amphiphilic structure; hence, they can penetrate easily into the bacterial cell membrane, leading to the disruption of metabolism [49]. Meanwhile, the antibacterial activity of the *W. trilobata* EOs may be attributed to its major components, which are mostly sesquiterpenes. The lipophilicity of sesquiterpenoids has been shown to lead the interaction with the cell membrane, and their accumulation may result in the impairment of the structural and functional level of bacterial cells [50].

Phenolic compounds are known as naturally occurring antioxidants, and their antioxidative properties also depend on the structure and number of hydroxyl groups. A higher number of hydroxyl groups present in the structure of phenolic compounds results in higher antioxidant properties, as long as there is no steric hindrance [51]. In addition, phenolic O-H bonds react quickly to DPPH radicals as well as peroxyl (ROO·) radicals [52]. The higher reactivity of DPPH may influence the antioxidant potency of quercetin used as a standard against DPPH compared to its capability to scavenge NO radicals. Quercetin exhibited high antioxidant activity against DPPH due to its ability to donate electron or undergoing hydrogen atom transfer. Conversely, NO, a diatomic molecule with a single unpaired electron generated from SNP, may display a slower reaction rate due to the indirect measurement of NO by nitrite production. Despite both DPPH and NO radical neutralization mechanisms involving the same single electron transfer or hydrogen atom transfer, various factors may affect the reaction, such as the nitrite content in the plant extracts. The high nitrite content of leaf extracts may interrupt the NO radical scavenging measurement, leading to lower antioxidant potency against NO. This possibility may contribute to the lower scavenging activity of NO radicals observed in most crude leaf extracts compared to DPPH radicals.

The TPC and TFC results were correlated with the antioxidant properties of *B. rotunda* and *C. sinensis* (Figure 2). The highest flavonoid content in *C. sinensis* hexane extract suggested its high antioxidant activity toward DPPH and NO radicals (Table 2). On the other hand, the *B. rotunda* hexane extract possessing relatively high antioxidant activities against DPPH and NO radicals had the highest TPC value of 24.33 ± 0.96 mg GAE/g dry extract compared with all the tested extracts (Figure 2A and Supplementary Table S2). However, we determined the TFC value of the *B. rotunda* extract (Figure 2B and Supplementary Table S3) to be low, indicating that the high antioxidant activity of this extract may be derived from the content of phenolic compounds that are not classified as flavonoid secondary metabolites. On the other hand, the hydro-distilled EOs from this plant species also showed relatively good NO radical scavenging activity. Among all the tested EOs, the *B. rotunda* EOs showed the highest inhibition values for DPPH and NO radical scavenging activities. However, the TPC and FRAP values were low compared with other tested samples.

Pinostrobin is known as a dietary flavonoid and can be found in various marketed functional food products (e.g., honey, bee propolis, *B. rotunda* tincture, *Alpinia galangal* tincture), which exhibit various pharmacologic activities including antiaromatase, antiviral, antioxidant, antimicrobial, anti-inflammatory, and antinociceptive properties [53]. Pinostrobin, one of the major bioactive compounds in the hexane extract of *B. rotunda*, has been successfully isolated using a nonchromatographic method with 1.12% yield. Supplementary Figures S4–S7 show all the characterization spectra of the isolated crystal. All spectroscopic data are identical with those in the previous literature, including ^1^H-NMR spectrum [54], GC–MS data [55], and UV–Vis spectrum [56]. A previous study reported that the isolation of pinostrobin using a non-chromatographic and precipitation technique yielded colorless crystals with 0.882% yield by precipitating with petroleum benzene during a slow cooling condition [57]. In comparison with both a previous study and the common column chromatography technique, our findings demonstrated our method to have a higher yield and be simpler, faster, and more cost-effective, implying its feasibility in upscale production with high purity, low energy consumption, and simple apparatus.

Based on the COX-2 inhibition results, *B. rotunda* EOs and hexane extract exhibited less than 50% inhibition of COX-2, while pinostrobin showed the potency to be as effective as diclofenac sodium and BD (Figure 3). The results implied that both *B. rotunda* extract and EOs as well as pinostrobin had a potency for suppressing skin inflammation mediated by pro-inflammatory prostanoids related to AD pathogenesis, such as PGE_2_ and PGD_2_, by the inhibition of COX-2. However, pinostrobin had inferior activity compared to indomethacin as an alternative COX-2 inhibitor with an IC_50_ value of 0.26 ± 0.03 µg/mL [35].

Even though the pinostrobin was inactive against *S. aureus* with an MIC value of >200 µg/mL (Table 1), it also exhibited potent anti-inflammatory activity toward human 5-LOX. In addition, pinostrobin possessed FRAP (Table 2), which suggests its potential to halt the iron bearing in the substrate-binding cavity of lipoxygenase enzyme in the ferric state. NDGA, as a redox-type inhibitor, has potent antioxidant activity to maintain the iron in the ferric state, interrupting the redox cycle of LOXs and leading to inactivation [58]. Compared to zileuton as a specific 5-LOX inhibitor (IC_50_ = 0.92 ± 0.21 µM) [59], pinostrobin also exhibited superior activity (IC_50_ = 0.499 ± 0.129 µM). Furthermore, we continued investigating this kinetic study and molecular modeling to study a mechanism of action towards 5-LOX.

According to the results, NDGA and pinostrobin were considered to be mixed-type inhibitor. NDGA is widely known as a redox-active inhibitor that is bound in the active site of 5-LOX, resulting in the capping helix alteration [42]. However, limited information is available about its probability to bind to the allosteric site of 5-LOX. Furthermore, there is no report about the pinostrobin inhibitory action to the 5-LOX. Molecular modeling was further carried out to support these findings.

The binding of (R)-pinostrobin to the 5-LOX binding site is more comparable to NGDA. The chromone ring of (R)-pinostrobin is oriented towards Gln363, Thr363, His432, and Arg596 residues, in which hydrogen bonds were formed, and nearby hydrophobic residues, Phe359, Trp599, and Pro569, are predicted to interact with the ligand by hydrophobic interactions. The phenyl ring is hydrophobic and was seen to occupy a hydrophobic region that can form hydrophobic interactions with Leu368. (S)-pinostrobin displayed a more distinctive binding mode. The carbonyl oxygen of the chromone ring is oriented to form a hydrogen bond with Arg596, permitting the methoxy group to form a hydrogen bond with Thr364. Vicinal hydrophobic residues, Phe359, Trp599, and Pro569, were predicted to form hydrophobic interactions with the 6 chromone ring, whereas Leu368 is predicted to form the same interaction with the phenyl ring.

The results from the molecular docking simulation are in line with the in vitro results and steady-state kinetic study of 5-LOX by pinostrobin and NDGA. Both compounds were shown to competitively bind to the active site of 5-LOX based on the kinetic study and molecular modeling. Furthermore, as the mixed-type inhibitors, both compounds were suggested to bind non-competitively on the allosteric site of 5-LOX. To the best of our knowledge, this study is the first report to elucidate the 5-LOX inhibitory activity of pinostrobin isolated from *B. rotunda* by an in vitro steady-state kinetic study and molecular modeling. Another finding reported the activity of (–)-pinostrobin towards another isomer of lipoxygenase (soybean 15-lipoxygenase), which was inactive at the tested range of concentrations. Furthermore, at a concentration of 25 µg/mL, (–)-pinostrobin inhibited NO production in LPS-induced RAW264.7 murine macrophage cells by 90.84% without a cytotoxic effect [60]. In addition, pinostrobin was reported to be nontoxic for dermal application. According to a previous study, pinostrobin inhibited keratinocyte (HaCaT) cells with IC_50_ and full maximal inhibitory concentration (IC_100_) values of 30.76 µg/mL and >250 µg/mL, respectively [61]. Furthermore, by topical application in a mouse model, no dermal toxicity was observed at a concentration of 1000 mg/kg (e.g., dermatosis, ulcers, erythema, eczema, dermatitis, and piloerection) [62]. These reports have supported the safety of pinostrobin as a topical 5-LOX and COX-2 inhibitor.

Oxidative stress and an imbalance of the status of indigenous antioxidants are factors that contribute to AD pathogenesis. The level of lipid peroxidation was reported to be increased in AD patients compared with healthy people [62]. Furthermore, a case–control study among young children revealed that a higher antioxidative nutritional status reduced the risk of AD [10]. Accordingly, the supplementation of natural antioxidants such as vitamins is suggested as an adjunct to conventional therapy [63,64]. The dominance of *S. aureus* on the skin microbiome of AD patients is also a factor that contributes to the elevation of oxidative stress that occurs in the AD skin condition caused by the reactive oxygen species released by monocytes to attack this pathogen [64].

This study discovered that *B. rotunda* used in traditional medicine had an efficacy for atopic dermatitis management as it possessed anti-inflammatory activities by COX-2 and 5-LOX inhibition, antioxidant potentials, and antibacterial activity against *S. aureus*. In particular, EOs of *B. rotunda* exhibited human 5-LOX inhibitory activity, which was equal to that of NDGA and BD. Furthermore, isolated pinostrobin demonstrated comparable COX-2 inhibitory activity with NSAIDs diclofenac sodium and corticosteroid BD, and it also showed more potency against human 5-LOX than NDGA and BD as standard anti-inflammatory drugs. An enzyme kinetic study and molecular modeling demonstrated the mixed-type inhibition of pinostrobin to 5-LOX. Because a multipharmacological action such as anti-inflammation, antioxidant, and antibacterial activity of active compounds derived from plants is a promising supplement for managing the AD skin condition, we suggest that pinostrobin and *B. rotunda* should be further tested and applied as either topical care or nutraceutical and functional foods since they are promising in attenuating AD-like symptoms [65]. Pinostrobin may substitute the use of corticosteroids in AD treatment or can be applied in a combination therapy with other anti-inflammatory drugs to reduce the administration dose and thereby minimize systemic side effects. For maintaining mild symptoms, pinostrobin and *B. rotunda* extract may be formulated into daily skin cares.

An in vitro study is necessary to obtain initial insights into pharmacological action for skin diseases. Nowadays, ex vivo cellular and tissue models are being used to evaluate the efficacy and safety of pharmaceutical and cosmetic research, providing reliable and speedy results in the laboratory [66]. Three-dimensional (3D) skin models by cell culture were developed for AD skin [1,67,68]. Further studies with human artificial skin or bioengineered artificial skin substitutes such as 3D cell culture reconstructed human epithelium are suggested to be conducted to study toxicity, skin penetration, corrosive properties, or skin irritation before conducting an in vivo study with animal models or in a human trial [66]. Other inflammatory markers such as cytokines and skin barrier improvement (e.g., filaggrin and involucrin determination) related to AD pathogenesis should also be evaluated. Eventually, we recommend that further studies are essentially needed to investigate potency and the underlying mechanisms of action of pinostrobin for AD therapy by in vivo studies and clinical trials.

## 5. Conclusions

Therapy for multifactorial AD remains clinically challenging. Supplementation with plant-derived natural products may be a promising strategy as a source of anti-inflammatory, anti-infective, wound-healing, skin barrier repair, and antioxidant properties related to AD therapy. Among all seven medicinal plants tested, both *B. rotunda* EOs and hexane extract showed promising multi-pharmacological properties, including antioxidative, anti-staphylococcal, and anti-inflammation, in an in vitro study. A dietary flavonoid pinostrobin isolated from the *B. rotunda* hexane extract showed dual potent COX-2 and 5-LOX inhibitory activities. This study is the first report to reveal that pinostrobin is a potent 5-LOX inhibitor that is more active than NDGA and BD and has a mixed-type inhibition mode, as shown by enzyme inhibition kinetic and molecular modeling studies. Both *B. rotunda* EOs and hexane extract and its pinostrobin are promising to be developed for AD therapy as either topical skincare treatments or nutraceuticals.

## Figures and Tables

**Table 1:** Antibacterial activity of all medicinal plants against *Staphylococcus aureus*.

No.	Species	Part Used	Zone of Inhibition (mm)		MIC (µg/mL)	
			Essential Oils (c = 1000 µg/Disk)	Hexane Extract (c = 250 µg/Disk)	Essential Oils	Hexane Extract
1	*A. vulgaris*	Aerial part	-	-	nd	nd
2	*B. rotunda*	Rhizome	13	9	100	10
3	*C. sinensis*	Young leaves	12	-	nd	nd
4	*C. fistula*	Leaves	nd	-	nd	nd
		Pods	nd	-	nd	nd
5	*D. longan*	Peels	-	-	nd	nd
		Seeds	nd	-	nd	nd
6	*M. villosa*	Leaves	-	-	nd	nd
7	*W. trilobata*	Leaves	9	-	nd	nd
Pinostrobin			nd		>200	
Tetracycline			nd		3.125	

Disk diameter: 6 mm. nd, not determined. Inactive at ≤6mm for zone of inhibition. Inactive at >200 µg/mL for MIC values.

**Table 2:** Antioxidant activities of all selected medicinal plants.

No.	Species	Part Used	DPPH Radical; IC_50_ (µg/mL)		NO Radical; IC_50_ (µg/mL)		FRAP (mM FeSO_4_/g Extract)	
			EOs	Hexane Extract	EOs	Hexane Extract	EOs	Hexane Extract
1	*A. vulgaris*	Aerial part	-	-	-	-	0.37 ± 0.05 ^ab^	0.36 ± 0.14 ^ab^
2	*B. rotunda*	Rhizome	4085.67 ± 545.13 ^d^	557.97 ± 27.86 ^a^	1695.00 ± 128.18 ^b^	2651.67 ± 138.68 ^d^	0.24 ± 0.08 ^a^	2.26 ± 0.17 ^h^
3	*C. sinensis*	Young leaves	-	208.90 ± 4.81 ^a^	-	1809.67 ± 91.22 ^bc^	0.51 ± 0.09 ^bc^	3.87 ± 0.16 ^j^
4	*C. fistula*	Leaves	nd	3993.67 ± 175.25 ^d^	nd	4620.33 ± 285.35 ^e^	nd	2.61 ± 0.20 ^i^
		Pods	nd	-	nd	-	nd	1.12 ± 0.13 ^f^
5	*D. longan*	Peels	-	1123.00 ± 115.21 ^b^	-	675.70 ± 54.37 ^a^	1.09 ± 0.11 ^ef^	1.13 ± 0.14 ^f^
		Seeds	nd	-	nd	-	nd	0.97 ± 0.08 ^def^
6	*M. villosa*	Aerial part	-	2885.33 ± 280.08 ^c^	-	-	0.99 ± 0.07 ^def^	1.41 ± 0.27 ^g^
7	*W. trilobata*	Leaves	-	-	2849.67 ± 82.80 ^d^	2434.33 ± 984.08 ^cd^	0.84 ± 0.09 ^de^	0.73 ± 0.22 ^cd^
Quercetin			19.08 ± 1.34 (63.13 ± 4.43 µM)		93.58 ± 7.93 (309.62 ± 26.24 µM)		-	-
Pinostrobin			>1000		>1000		1.04 ± 0.01	

Inactive at concentration > 5000 µg/mL for DPPH and NO assays. nd, not determined. One-way ANOVA was performed to compare the mean of each sample in each assay, and different letters in each assay indicate the significant difference in the mean.

## Data Availability

The data that support the findings of this study are available in the manuscript and Supplementary Information.

## Supplementary Materials

The following supporting information can be downloaded at: https://www.mdpi.com/article/10.3390/antiox13010074/s1, Table S1: List of selected medicinal plants and percentage yields of their nonpolar fractions (essential oils and hexane extracts); Table S2: Determination of total phenolic content (TPC) values of all selected plants; Table S3: Determination of total flavonoid content (TFC) values of all selected plants; Table S4: Chemical composition of volatile compounds of essential oils and hexane extract derived from *B. rotunda*; Table S5: Chemical composition of volatile compounds in *C. sinensis* essential oils detected by GC–MS analysis; Table S6: Chemical composition of volatile compounds in *W. trilobata* essential oils detected by GC–MS analysis; Table S7: Determination of IC_50_ values of pinostrobin derived from *B. rotunda* against human cyclooxygenase-2; Table S8: Determination of IC_50_ vaules of EOs, hexane extracts, and pinostrobin derived from *B. rotunda* against human 5-lipoxygenase; Table S9: Calculated kinetic parameter values of pinostrobin compared to NDGA by the mixed-type inhibition; Table S10: Human recombinant 5-lipoxygenase velocity and Michaelis–Menten constant in the presence of NDGA and pinostrobin; Figure S1: Gas Chromatography (GC) chromatograms of (A) hexane extract and (B) EOs of *B. rotunda* and their major volatile components (C): camphene (C_1_), 1.8-cineole (C_2_), β-cis-ocimene (C_3_), camphor (C_4_), nerol (C_5_), methyl cinnamate (C_6_), and pinostrobin (C_7_); Figure S2: GC chromatogram of essential oils derived from *C. sinensis*; Figure S3: GC chromatogram of essential oils derived from *W. trilobata*; Figure S4: Thin-layer chromatography of isolated pinostrobin (P) and hexane extract derived from *B. rotunda* (UAE BR) developed in a mobile phase system containing a co-solvent mixture of n-hexane:ethyl acetate (0.5:9.5 *v*/*v*) and visualization under ultraviolet lights (254 nm; green and 366 nm; blue); Figure S5: UV–Vis spectrum of pinostrobin (in methanol) at the maximum absorbance (285.5 nm); Figure S6: Mass spectrometry fragmentation pattern of pinostrobin using gas chromatography–mass spectrometry (GC–MS) analysis with a DSQ quadrupole mass analyzer and an electron impact (EI) ionization positive mode at 70 eV; Figure S7: ^1^H-NMR spectrum of pinostrobin (in acetone-d6); Figure S8: Superimposition of the lowest energy conformer of docked nordihydroguaiaretic acid (NGDA) and the co-crystallized NGDA in the 5-lipoxygenase binding site.

## Abbreviations

H-NMR: ^1^H-nuclear magnetic resonance; 5-LOX: 5-lipoxygenase; AD: atopic dermatitis; ANOVA: analysis of variance; BD: betamethasone dipropionate; COX-2: cyclooxygenase-2; DPPH: 2,2-diphenyl-1-picrylhydrazyl; EOs: essential oils; FLAP: 5 lipoxygenase-activating protein; FRAP: ferric reducing antioxidant power; GC–MS: gas chromatography–mass spectrometry; IC_50_: half-maximal inhibition concentration; K_i_: inhibitor constant; K_m_: Michael constant; LA: Luria agar; LB: Lysogeny broth; LTB_4_: Leukotriene B_4_; LTC_4_: Leukotriene C_4_; MIC: minimum inhibitory concentration; NDGA: nordihydroguaiaretic acid; NED: *N*-(1-naphtyl)-ethylenediamine; NO: nitric oxide; PBS: phosphate-buffered saline; PGD_2_: Prostaglandin G_2_; PGE_2_: Prostaglandin E_2_; PGG_2_: Prostaglandin G_2_; RT: retention time; SNP: sodium nitroprusside; TCM: traditional Chinese medicine; TFC: total flavonoid content; Th2: T helper 2 cells; TPC: total phenolic content; UAE: ultrasound-assisted extraction; V_max_: maximum velocity.
