# Supplementary material for: Boesenbergia rotunda and Its Pinostrobin for Atopic Dermatitis: Dual 5-Lipoxygenase and Cyclooxygenase-2 Inhibitor and Its Mechanistic Study through Steady-State Kinetics and Molecular Modeling
Source: Antioxidants (Basel). 2024 Jan 5;13(1):74. doi: 10.3390/antiox13010074 (PMC10812521; doi:10.3390/antiox13010074)
Supplement: Supplementary file 1 [file antioxidants-13-00074-s001.zip › antioxidants-2745411-supplementary.pdf]

Supplementary Information

Boesenbergia rotunda and its pinostrobin for atopic dermatitis: Dual 5-lipoxygenase and cyclooxygenase-2 inhibitor and its mechanistic study through steady-state kinetics and molecular modeling

Desy Liana[1], Chatchakorn Eurtivong[2], and Anuchit Phanumartwiwath[1*]

[a]College of Public Health Sciences, Chulalongkorn University, Bangkok 10330, Thailand.

[b]Department of Pharmaceutical Chemistry, Faculty of Pharmacy, Mahidol University, Ratchathewi, Bangkok 10400, Thailand.

*Corresponding author: Dr. Anuchit Phanumartwiwath; E-mail: anuchit.p@chula.ac.th

1. **Extraction yield (%) of essential oils and hexane extracts derived from the selected medicinal plants**

Table S1. List of selected medicinal plants and percentage yields of their nonpolar fractions (essential oils and hexane extracts).

No.	Plant species	Part used	Essential oils		Hexane extracts	
			Yield (%)	Characteristics	Yield (%)	Characteristics
1	*A. vulgaris* L.	Aerial part	0.183	Yellow clear liquid	4.01	Crude extract
2	*B. rotunda* (L.) Mansf.	Rhizome	0.216	Yellowish clear liquid	2.43	Oily crude extract
3	*C. sinensis* (L.) Kuntze	Young leaves	0.016	Yellowish waxy liquid	5.74	Crude extract
4	*C. fistula* L.	Leaves	-	-	2.37	Crude extract
		Pods	-	-	0.63	Crude extract
5	*D. longan* Lour.	Peel	0.013	Yellowish liquid	0.44	Crude extract
		Seed	-	-	2.19	Fixed oils
6	*M. villosa* Huds.	Aerial part	0.012	Cloudy, waxy greenish liquid	1.58	Crude extract
7	*W. trilobata* (L.) Hitchc.	Leaves	0.086	Greenish clear liquid	2.53	Crude extract

2. Total phenolic content of all plant extracts

Table S2. Determination of total phenolic content (TPC) values of all selected plants.

No	Sample	TPC value (mg gallic acid/g dry weight of extract)			
		1	2	3	Mean ± SD
1	UAE BR	24.567	23.280	25.158	24.335 ± 0.960[i]
2	EO MV	20.184	21.610	19.628	20.474 ± 1.023[h]
3	UAE AV	16.428	18.584	16.045	17.019 ± 1.369[gh]
4	UAE CSD	17.610	16.845	15.315	16.590 ± 1.169[g]
5	UAE DLP	15.697	15.419	15.489	15.535 ± 0.145[f]
6	EO DLP	11.419	11.558	10.097	11.025 ± 0.806[e]
7	UAE MV	9.228	8.671	7.419	8.439 ± 0.926[d]
8	EO BR	6.967	6.480	6.306	6.584 ± 0.343[c]
9	UAE WT	6.306	4.984	5.193	5.494 ± 0.711[bc]
10	UAE CFF	4.184	4.010	5.019	4.405 ± 0.960[b]
11	EO WT	0.650	0.758	0.758	0.722 ± 0.062[a]
12	UAE CFL	0.483	0.574	0.591	0.550 ± 0.058[a]
13	UAE DLS	0.289	0.313	0.376	0.326 ± 0.045[a]
14	EO AV	0.268	0.344	0.299	0.304 ± 0.038[a]
15	EO CSD	0.268	0.351	0.282	0.300 ± 0.046[a]

*Note: UAE: hexane extract; EO: essentail oils; BR: *Boesenbergia rotunda*; MV: *Mentha villosa*; AV: *Artemisia vulgaris*; CSD: *Camellia sinensis*; DLP: *Dimocarpus longan* peels; DLS: *Dimocarpus longan* seeds; CFF: *Cassia fistula* fruits; WT: *Wedelia trilobata;* and CFL: *Cassia fistula* leaves. One-way ANOVA was performed to compare the mean of each sample, and different letters indicate the significant difference of the mean.

3. Total flavonoid content of all plant extracts

Table S3. Determination of total flavonoid content (TFC) values of all selected plants.

No	Sample	TFC value (mg quercetin/g dry weight of extract)			
		**1**	**2**	**3**	**Mean ± SD**
1	UAE CSD	105.851	109.957	101.904	105.904 ± 4.027[d]
2	UAE MV	41.264	42.384	41.957	41.868 ± 0.565[c]
3	UAE CFL	38.171	38.437	35.931	37.513 ± 1.377[b]
4	UAE CFF	5.744	6.171	5.317	5.744 ± 0.427[a]
5	UAE AV	5.531	5.477	5.317	5.442 ± 0.111[a]
6	UAE WT	5.157	5.584	5.264	5.335 ± 0.222[a]
7	UAE BR	4.304	3.824	4.304	4.144 ± 0.244[a]
8	UAE DLP	3.131	3.557	3.291	3.326 ± 0.145[a]

*Note: UAE: hexane extract; CSD: *C. sinensis*; MV: *M. villosa*; CFL: *C. fistula* leaves; CFF: *C. fistula* fruits; AV: *A. vulgaris*; WT: *W. trilobata*; BR: *B. rotunda*; and DLP: *D. longan* peels. One-way ANOVA was performed to compare the mean of each sample, and different letters indicate the significant difference of the mean.

4. **Gas chromatography–mass spectrometry of *Boesenbergia rotunda* essential oils and hexane extracts**

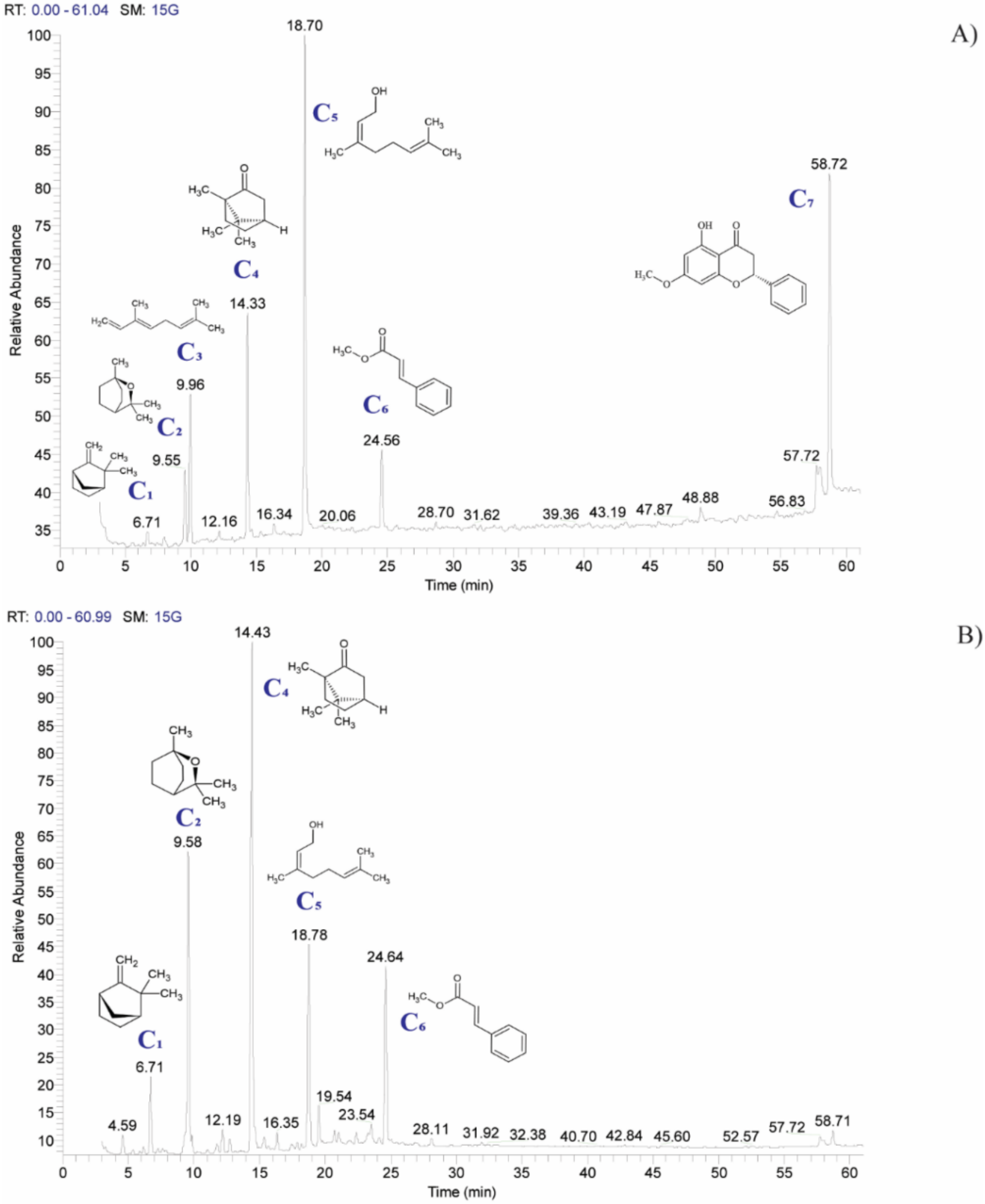

Figure S1. Gas Chromatography (GC) chromatograms of A). hexane extract and B). EOs of *B. rotunda*, and their major volatile components (C): camphene (C_1), 1.8-cineole (C_2), β-cis-ocimene (C_3), camphor (C_4), nerol (C_5), methyl cinnamate (C_6), and pinostrobin (C_7).

Table S4. Chemical composition of volatile compounds of essential oils and hexane extract derived from *B. rotunda*.

No.	RT* (min)	Compound	% Abundance EOs	Hexane extract
1	4.59	2-Hexenal	1.30	-
2	6.71	Camphene	4.97	1.57
3	9.58	1,8-cineole	20.47	4.80
4	9.96	β-cis-ocimene	-	8.77
5	12.19	Linalool	1.36	1.09
6	14.43	Camphor	35.25	13.99
7	15.36	Borneol	1.36	-
8	16.35	α-terpineol	1.06	1.17
9	18.78	Nerol	13.86	32.76
10	19.54	Citral	2.92	-
11	22.38	cis-Limonene oxide	0.69	-
12	23.54	Limonene oxide	2.36	-
13	24.64	Methyl cinnamate	11.71	5.46
14	28.7	(E) α-Bergamotene	-	0.88
15	48.88	(Z) 6-Pentadecen-1-ol	-	1.50
16	57.72	(Z) 9-Octadecenamide	1.34	6.79
17	58.72	Pinostrobin	1.33	21.22

*RT based on the GC chromatogram of essential oils derived from *B. rotunda*.

5. Gas chromatography–mass spectrometry of *Camellia sinensis* essential oils

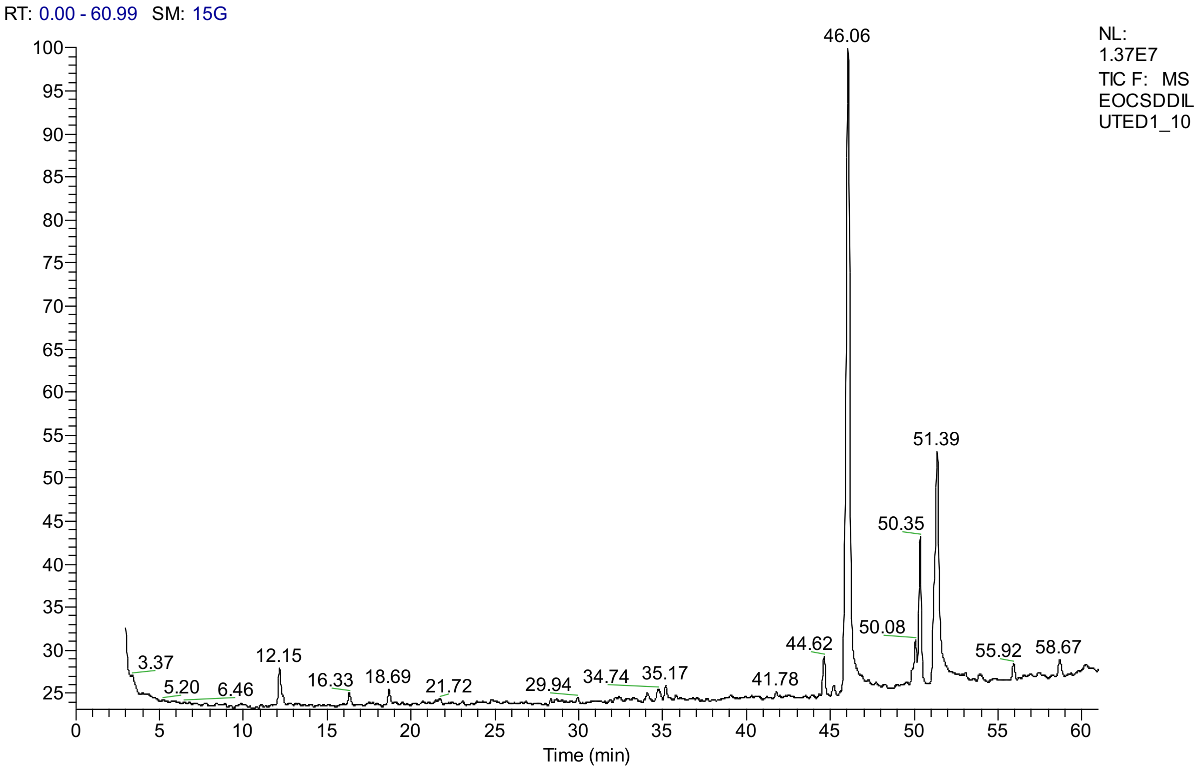

Figure S2. GC chromatogram of essential oils derived from *C. sinensis*.

Table S5. Chemical composition of volatile compounds in *C. sinensis* essential oils detected by GC–MS analysis.

No	RT (min)	% Abundance	R Match	Compound
1	12.15	2.72	898	Linalool
2	16.33	1.21	870	α-terpineol
3	18.69	1.37	829	Nerol
4	20.7	0.33	787	2,4-Decadienal
5	21.72	0.79	835	Bicyclo [2.1.0] pentane, 1,4-dimethyl-
6	23.06	0.26	900	Naphthalene, 1,2-dihydro-4,5,7-trimethyl-
7	24.82	0.35	854	Phenol, 4-(1,1-dimethylpropyl)-
8	28.32	0.36	847	β-ionone (E)
9	29.94	0.351	824	Calamenene (Z)
10	34.1	0.73	835	Cubenol
11	34.74	1.04	895	Murrolol <epi-alpha->
12	35.17	0.98	937	Murrolol <epi-alpha->
13	35.8	0.48	809	Cadalene
14	44.62	2.37	904	Methyl hexadecanoate
15	46.06	52.54	903	n-hexadecanoic acid
16	50.35	11.28	876	Phytol
17	51.39	21.98	901	Linolenic acid, methyl ester
18	55.92	0.85	856	Tricosane

6. Gas chromatography–mass spectrometry of *Wedelia trilobata* essential oils

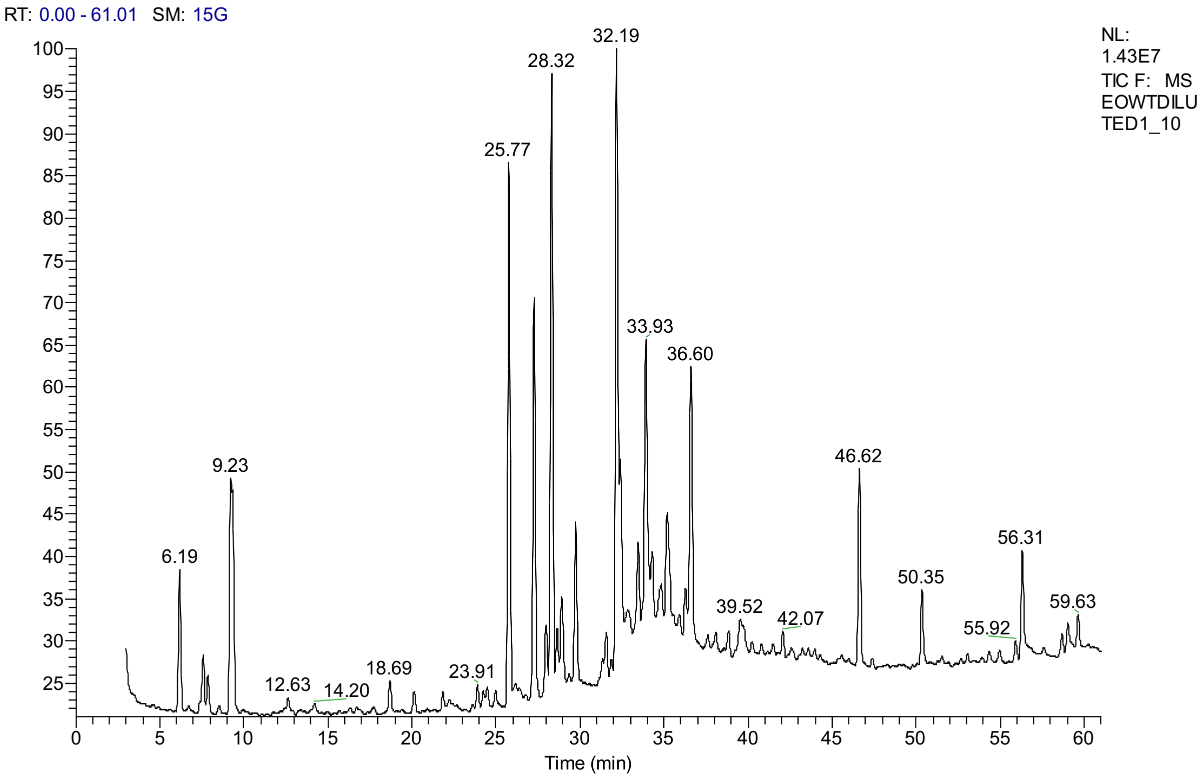

Figure S3. GC chromatogram of essential oils derived from *W. trilobata*.

Table S6. Chemical composition of volatile compounds in *W. trilobata* essential oils detected by GC–MS analysis.

No	RT (min)	% Abundance	R Match	Compound
1	6.19	2.51	944	α-pinene
2	6.71	0.21	887	Camphene
3	7.58	1.25	940	(-)-β-Pinene
4	8.53	0.18	861	α-Phellandrene
5	9.23	7.50	911	o-Cymene
6	12.63	0.73	798	Perillene
7	18.69	0.69	865	Eucarvone
8	20.14	0.44	919	Bornyl acetate
9	21.84	0.37	788	Spiro [4.5] decan-6-one
10	23.91	0.63	918	α-copaene
11	24.47	0.87	916	α-guaiene
12	25.77	9.88	962	β-caryophyllene(E-)
13	27.27	7.63	935	α-humulene
14	28.32	12.51	925	Germacrene D
15	28.9	3.09	917	γ-elemene
16	29.75	3.37	916	δ-cadinene
17	31.55	1.34	917	Nerolidol (E-)
18	32.19	16.98	936	Spathulenol
19	33.46	1.67	780	Caryophyllene oxide
20	33.93	7.89	932	Junenol
21	35.19	3.53	901	Murrolol<epi-alpha->
22	36.6	6.69	851	Carotol
23	37.62	0.35	710	Spathulenol
24	38.09	0.59	840	Oplopanone
25	46.62	3.73	792	Androst-2,16-diene
26	50.35	1.77	838	Phytol
27	55.92	0.35	896	Tricosane
28	56.31	2.74	927	Grandiflorenic acid
29	58.67	0.50	893	Eicosane

7. Characterization of pinostrobin isolated from *Boesenbergia rotunda*

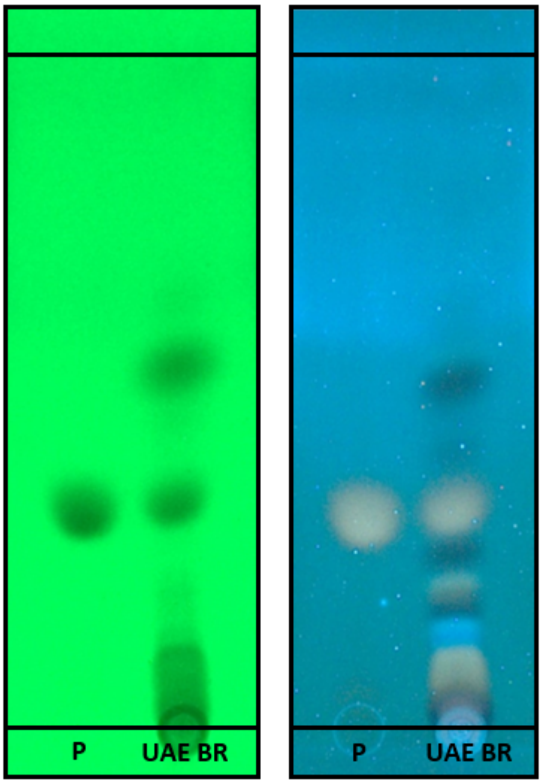

Figure S4. Thin layer chromatography of isolated pinostrobin (P) and hexane extract derived from *B. rotunda* (UAE BR) developed in a mobile phase system containing a co-solvent mixture of *n*-hexane:ethyl acetate (9.5:0.5 v/v) and visualization under ultraviolet lights (254 nm; green and 366 nm; blue).

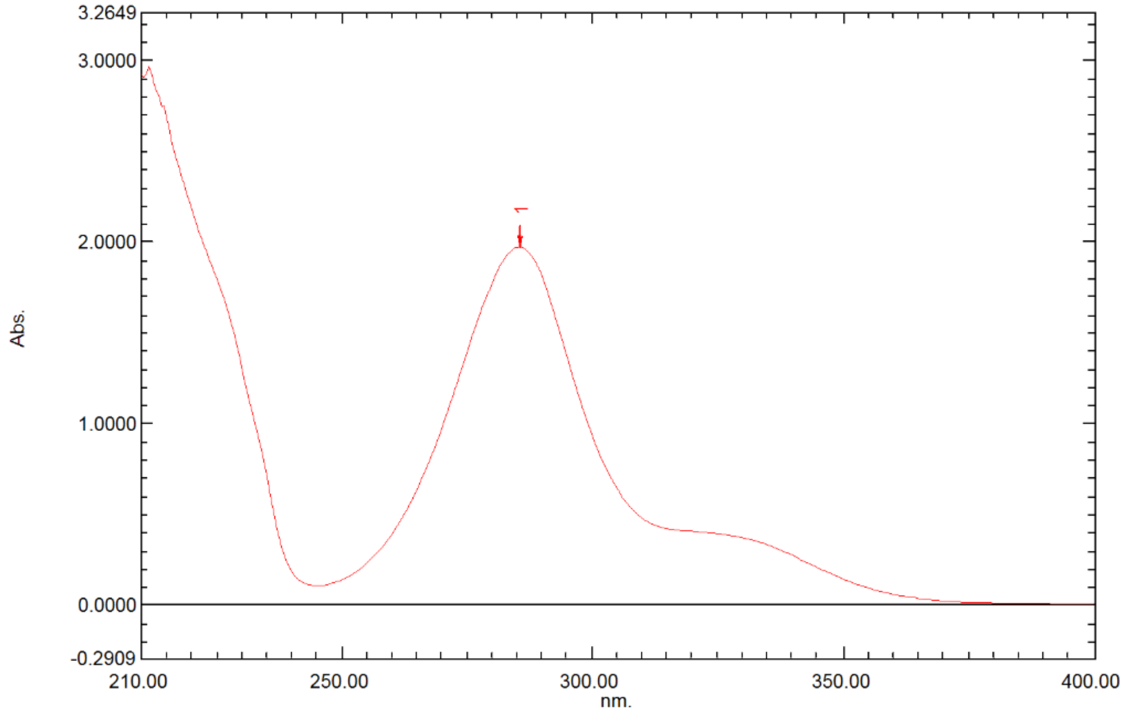

Figure S5. UV–Vis spectrum of pinostrobin (in methanol) at the maximum absorbance (285.5 nm).

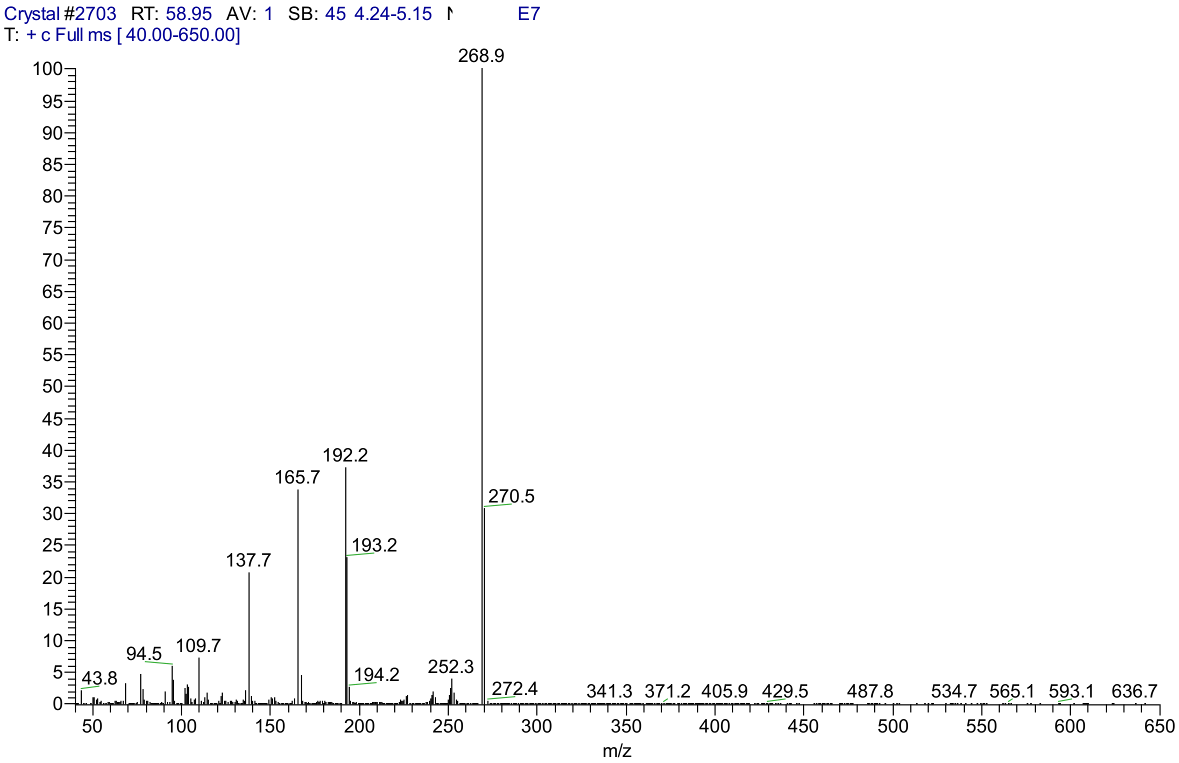

Figure S6. Mass spectrometry fragmentation pattern of pinostrobin using gas chromatography–mass spectrometry (GC–MS) with a DSQ quadrupole mass analyzer and an electron impact (EI) ionization positive mode at 70 eV.

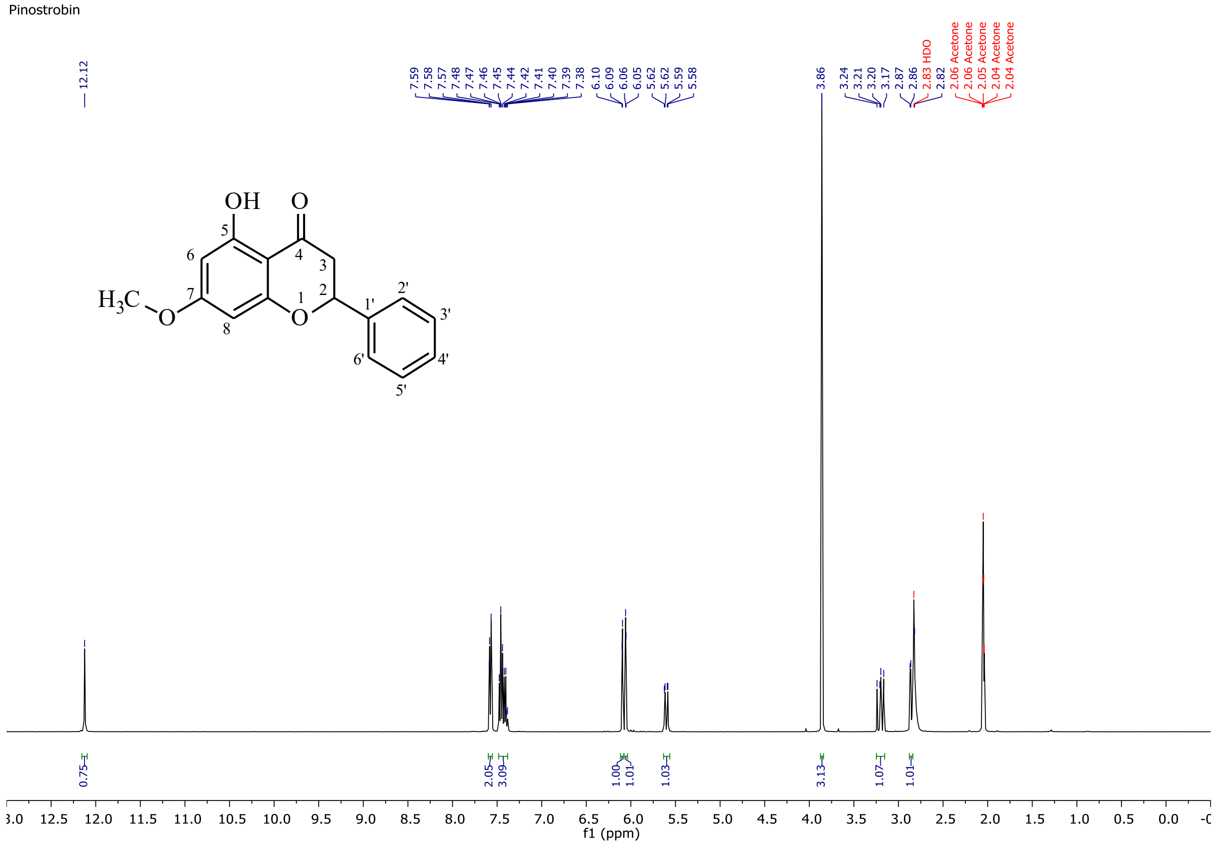

^{1}H NMR (400 MHz, Acetone-d_6): δ 12.12 (1H, s, 5-OH), 7.59 – 7.57 (2H, m, H-2′, H-6′), 7.48 – 7.38 (3H, m, H-3′, H-4′, H-5′), 6.10 (1H, d, *J* = 2.3 Hz, H-8), 6.06 (1H, d, *J* = 2.3 Hz, H-6), 5.60 (1H, dd, *J* = 3.1, 12.8 Hz, H-2), 3.86 (3H, s, 7-OCH_3), 3.20 (1H, dd, *J* = 12.8, 17.1 Hz, H-3_{ax}), 2.85 (1H, dd, overlapping, H-3_{eq}).

Figure S7. ^{1}H-NMR spectrum of pinostrobin (in acetone-d6).

8. **Cyclooxygenase-2 inhibitory activity (IC_{50}, μM) of essential oils, hexane extracts, and pinostrobin derived from *B. rotunda***

Table S7. Determination of IC_{50} values of pinostrobin derived from *B. rotunda* against human cyclooxygenase-2.

No	Samples	IC_{50} (μM)			
		1	2	Mean ± SD	Duncan
1	Pinostrobin	255.25	316.12	285.67 ± 43.03	a
4	Diclofenac sodium*	318.74	261.96	290.35 ± 40.14	a
5	BD**	248.31	231.86	240.09 ± 11.63	a

*Diclofenac sodium is a positive control.
**BD: betamethasone dipropionate is an atopic dermatitis standard drug.

9. **5-Lipoxygenase inhibitory activity (IC_{50}, μg/mL) of essential oils, hexane extracts, and pinostrobin derived from *B. rotunda***

Table S8. Determination of IC_{50} vaules of EOs, hexane extracts, and pinostrobin derived from *B. rotunda* against human 5-lipoxygenase.

No	Samples	IC_{50}, μg/mL						
		1	2	3	4	5	Mean ± SD	Duncan
1	Pinostrobin	0.147	0.101	0.098	0.179	0.149	0.135 ± 0.035	a
2	UAE BR	11.390	12.020	11.600	12.910	9.078	11.399 ± 1.423	c
3	EO BR	1.763	1.449	1.432	1.732	1.682	1.612 ± 0.159	b
4	NDGA**	1.256	1.458	1.210	1.730	1.936	1.518 ± 0.311	b
5	BD***	0.847	0.936	0.895	1.315	1.246	1.048 ± 0.216	b

*Note: UAE: hexane extract; EO: essentail oils; and BR: *B. rotunda*; five replicate wells were generated from two rounds of testing. One-way ANOVA was performed to compare the mean of each sample, and different letters indicate the significant difference of the mean.
**NDGA: nordihydroguaiaretic acid is a positive control.
***BD: betamethasone dipropionate is an atopic dermatitis standard drug.

10. Steady-state kinetics of 5-lipoxygenase in the presence of inhibitor NDGA and pinostrobin

Table S9. Calculated kinetic parameter values of pinostrobin compared to NDGA by the mixed-type inhibition.

Mixed-type Inhibition	NDGA	Pinostrobin
V_{max} (M/s)	7.888×10^{-6}	8.064×10^{-6}
K_m (μM)	3.919	3.249
K_i (μM)	3.376	2.284

*Note: NDGA = nordihydroguaiaretic acid; V_{max} = maximum enzyme velocity; K_m = Michaelis-Menten constant; K_i = inhibition constant.

Table S10. Human recombinant 5-lipoxygenase velocity and Michaelis-Menten constant in the presence of NDGA and pinostrobin.

Substrate concentration (μM)	Control (no inhibitor)	NDGA (μg/mL) 1.56	NDGA (μg/mL) 3.125	NDGA (μg/mL) 6.25	Pinostrobin (μg/mL) 1.56	Pinostrobin (μg/mL) 3.125	Pinostrobin (μg/mL) 6.25
66.67	0.0000090934	0.00000581159	0.0000060934	0.00000637037	0.00000548	0.00000548	0.00000559
33.335	0.0000077053	0.00000374396	0.0000044203	0.00000508776	0.00000375	0.00000417	0.00000423
16.6675	0.0000064549	0.00000277295	0.0000035853	0.00000395089	0.00000293	0.00000325	0.00000333
8.33375	0.0000065628	0.00000195652	0.0000035878	0.00000397907	0.00000262	0.00000297	0.00000311
**V_{max} (M/s)**	9.068×10^{-6}	8.695×10^{-6}	6.364×10^{-6}	6.662×10^{-6}	6.477×10^{-6}	6.073×10^{-6}	6.097×10^{-6}
**K_m (μM)**	4.259	36.40	9.402	7.823	17.44	11.60	10.85

*Note: NDGA = nordihydroguaiaretic acid; V_{max} and K_m were calculated using the Michaelis-Menten formula in GraphPad Prism v.9.

11. Molecular modeling of NDGA to human 5-lipoxygenase (PDB ID: 6N2W)

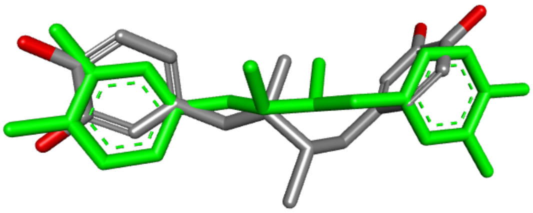

Figure S8. Superimposition of the lowest energy conformer of docked nordihydroguaiaretic acid (NGDA) and the co-crystallized NGDA in the 5-lipoxygenase binding site.
